# Molecular Mechanisms of Pharmaceutical Drug Binding into Calsequestrin

**DOI:** 10.3390/ijms131114326

**Published:** 2012-11-06

**Authors:** Arun K. Subra, Mark S. Nissen, Kevin M. Lewis, Ashwin K. Muralidharan, Emiliano J. Sanchez, Hendrik Milting, ChulHee Kang

**Affiliations:** 1 Department of Chemistry, Washington State University, Pullman, WA 99164-4630, USA; E-Mails: subramanianarunk@gmail.com (A.K.S.); mnissen@wsu.edu (M.S.N.); kevin.lewis@email.wsu.edu (K.M.L.); ashwin.murali@email.wsu.edu (A.K.M.); 2 School of Molecular Biosciences, Washington State University, Pullman, WA 99164-4660, USA; E-Mail: emiliano.j.sanchez@gmail.com; 3 Herz- und Diabeteszentrum NRW, Klinik der Ruhr Universitaet Bochum, Erich and Hanna Klessmann-Institut für Kardiovaskuläre Forschung und Entwicklung, 32545 Bad Oeynhausen, Germany; E-Mail: HMilting@hdz-nrw.de

**Keywords:** CASQ, calsequestrin, SR, sarcoplasmic reticulum, CASQ2, cardiac calsequestrin, CASQ1, skeletal calsequestrin, TFP, trifluoperazine, ITC, isothermal titration calorimetry, daunomycin, DAN, desipramine, DSP, diltiazem, DTZ, propafenone, PFN, thioridazine, TDZ

## Abstract

Calsequestrin (CASQ) is a major Ca^2+^-storage/buffer protein present in the sarcoplasmic reticulum of both skeletal (CASQ1) and cardiac (CASQ2) muscles. CASQ has significant affinity for a number of pharmaceutical drugs with known muscular toxicities. Our approach, with *in silico* molecular docking, single crystal X-ray diffraction, and isothermal titration calorimetry (ITC), identified three distinct binding pockets on the surface of CASQ2, which overlap with 2-methyl-2,4-pentanediol (MPD) binding sites observed in the crystal structure. Those three receptor sites based on canine CASQ1 crystal structure gave a high correlation (*R*^2^ = 0.80) to our ITC data. Daunomycin, doxorubicin, thioridazine, and trifluoperazine showed strong affinity to the S1 site, which is a central cavity formed between three domains of CASQ2. Some of the moderate-affinity drugs and some high-affinity drugs like amlodipine and verapamil displayed their binding into S2 sites, which are the thioredoxin-like fold present in each CASQ domain. Docking predictions combined with dissociation constants imply that presence of large aromatic cores and less flexible functional groups determines the strength of binding affinity to CASQ. In addition, the predicted binding pockets for both caffeine and epigallocatechin overlapped with the S1 and S2 sites, suggesting competitive inhibition by these natural compounds as a plausible explanation for their antagonistic effects on cardiotoxic side effects.

## 1. Introduction

Clinical usefulness of many effective drugs is often limited by their toxicity as a consequence of dose- and time-dependent accumulation within muscle tissue [1]. A wide spectrum of possible mechanisms of those drug-induced muscle toxicities have been proposed, including muscle enzyme defects; changes in mitochondrial function and intracellular metabolism; the influence on membrane stability; and interactions with *P*-glycoprotein or Cyt P450 [2,3]. However, the precise pathophysiologic and biochemical mechanisms of most drug-induced muscle toxicities remain unclear. The main SR proteins responsible for Ca^2+^ release process of the excitation-contraction coupling in cardiac and skeletal muscle are the Ca^2+^ release channel (RyR), junctin, triadin and the Ca^2+^-storage/buffer protein, calsequestrin (CASQ) [4]. There are several reports on the affinities of various drugs to RyR and their effects upon binding, such as dantrolene and caffeine [5–7]. In addition, both the skeletal and cardiac isoforms of CASQ (CASQ1 and CASQ2, respectively) show significant affinity for several classes of compounds such as anthracyclines, phenothiazines, and tricyclic antidepressants [8–10]. Many of those drugs can deposit in the sarcoplasmic reticulum (SR) and significantly perturb its critical role of storage and/or release of Ca^2+^ ions [10,11]. The structures of both CASQ1 and CASQ2 are composed of three thioredoxin-like domains [12–15], which, coincidently, is a binding motif for small molecules in many proteins [16,17]. Considering the high concentration of CASQ in muscle tissue (~100–500 mg mL^−1^), long-term or high-dose administration of those drugs could lead to their accumulation in the SR, despite their moderate affinity for CASQ (1–50 μM), which could substantially affect the Ca^2+^ content of the SR due to decreased buffering/storage capacity of CASQ brought upon by its binding of these drugs [9,10]. In particular, those compounds or their metabolites could affect patients who already suffer from compromised drug metabolism and clearance or from impairments in proper CASQ function, such as those afflicted with catecholaminergic polymorphic ventricular tachycardia (CPVT) or malignant hyperthermia (MH) [9,18].

As has been noticed for flavonoids [19] and PDE-5 inhibitors [20], caffeine has been known to improve the survival rate of cancer patients, probably by modulating the toxic side effects of anthracycline-derivatives like daunomycin (DAN) and doxorubicin (DOX) [21]. In addition, epigallocatechin (EGC) and epigallocatechin-3-gallate (EGCG) have been reported to reverse DOX-induced intra-luminal Ca^2+^-depletion in the SR [22]. In this study, we applied molecular docking, isothermal calorimetry, and crystallographic techniques to find the potential binding sites. The structural mechanism behind the apparent affinities of CASQ for several drug molecules and a structural model that explains the potential antagonistic effect of CAF and EGC were investigated through those comprehensive approaches.

## 2. Results and Discussion

### 2.1. Development of a Highly-Predictive Molecular Docking Model for CASQ2-Ligand Binding

**Figure 1:**
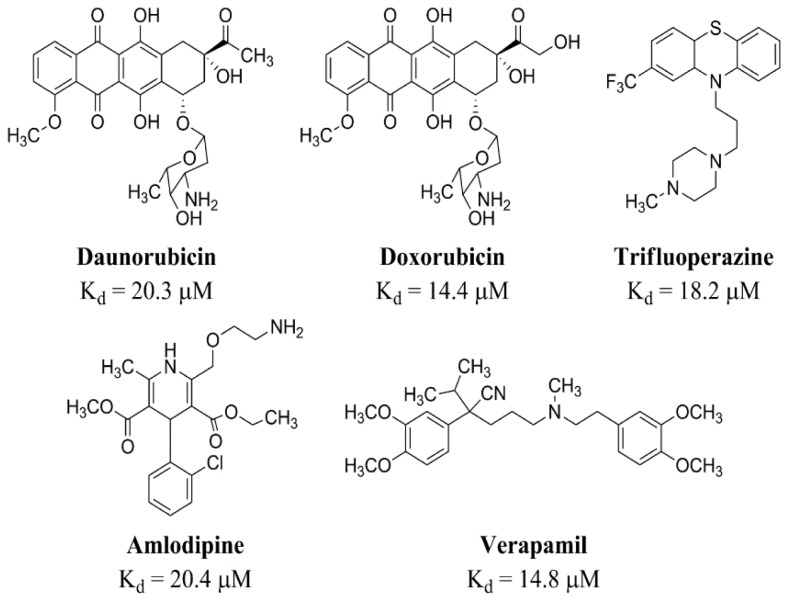
The 2-D line drawings of the strong binders for human cardiac muscles (hCASQ2) and their corresponding experimental dissociation constants (*K*_d_). Chemical structures for the complete set of the tested strong, intermediate, and poor binders are included in the Supplemental file 1.

We created an initial receptor model using the crystal structure of human CASQ2 (hCASQ2, PDB ID: 2VAF) to correlate the docking scores of drug compounds with their reported *K*_d_ values from previous ITC studies [9]. For this purpose, we used a blind docking approach by including the whole protein in the affinity grid because the location of ligand-binding pockets in CASQ is unknown. The studied drugs were grouped into the following three binding classes based on their ITC dissociation constants (*K*_d_) (Figure 1): strong, moderate, and poor. The strong binders were amlodipine (AMD), daunomycin (DAN), doxorubicin (DOX), thioridazine (TDZ), trifluoperazine (TFP), and verapamil (VPM) the moderate binders were amitriptyline (AMT), diltiazem (DTZ), imipramine (IMP), nortriptyline (NTP), and promethazine (PMT); and the poor binders were chlorpromazine (CPM), ephedrine (EPH), isoproterenol (IPT), metoprolol (MTP), miconazole (MNZ), and propafenone (PFN). The competitive inhibitors, caffeine (CAF), epigallocatechin (EGC), and epigallocatechin gallate (EGCG), were also studied along with the known hCASQ2 binding drugs.

**Figure 2:**
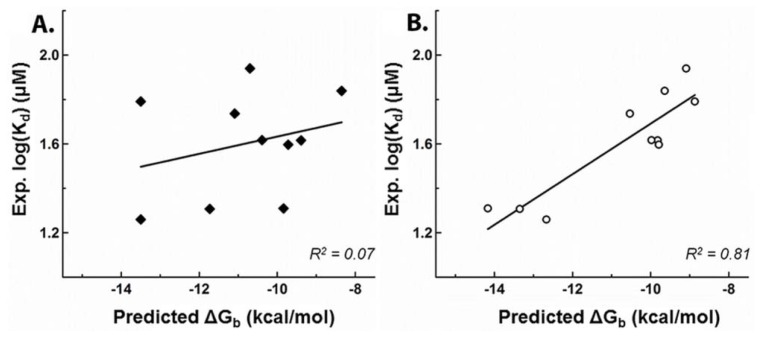
The 2-D correlation plots of the molecular docking scores (Δ*G*_b_) *versus* the logarithm of experimental dissociation constants, Experimental log(*K*_d_) through least-square fitting using the crystal structures of hCASQ2 (2VAF-left, **A**) and cCASQ2 (1SJI-right, **B**) as receptor models.

But this *in silico* model yielded a poor correlation between docking scores and experimental *K*_d_’s (*R*^2^ = 0.07), most likely due to potential inaccuracies in the side-chain conformations (Figure 2A) inherent to the low-resolution (3.8 Å) of the protein structure. The same docking procedure was then repeated using the 2.4 Å (PDB ID: 1SJI) resolution crystal structure of canine CASQ2 (cCASQ2) in order to mitigate the effects of uncertainties in those side-chain conformations/orientations. This significantly improved the correlation for drugs with strong or moderate affinities for hCASQ2 (*R*^2^ = 0.8) (Figure 2B). However, correlating the scores of poor binders with their corresponding experimental *K*_d_’s remained a challenge, as many of their scores were elevated to the moderate binding range, with some elevated to that of strong binders. Although the poor binders could not be correlated to the experiment, they were predicted to bind in the same pockets as those of strong and moderate binders. Visual analysis revealed less possibility for the presence of unique binding pockets for poor binders. This suggests that the bias in predictions might be coming from the scoring function instead, which could be over-predicting the binding interactions of poor binders. Inspection of the docking results for these poor binders highlighted that their aromatic cores were significantly smaller than those of moderate and strong binders, and possessed long highly flexible side chains with charges (Supplemental file 1). Hence, the large aromatic cores, flexibility of the side-chains and their sizes could be the crucial chemical features required for a tight fit into the CASQ2 binding pockets. Therefore, we excluded the docking predictions for poor binders on assumption that they suffer some docking bias and only focused on the strong and moderately binding molecules that could be well correlated to experimental *K*_d_ values.

### 2.2. Prediction of Small Molecule Binding Pockets in CASQ2

Although binding of several pharmaceutical drugs and their side effects on both Ca^2+^-binding capacity and polymerization of CASQ have been well established [9,10], their binding sites and the underpinning molecular mechanism of those toxic effects have been yet unknown. CASQ2 possesses several indentations of different sizes and charges in its highly negative surface. The majority of these pockets were hydrophobic while a large central junctional pocket was surrounded by charged amino acids. Based on their sizes, it was difficult to focus on one particular pocket because many of them indeed were suitable for binding of pharmaceutical compounds. Based on the presence of plausible pockets, we hypothesized that there could be differential affinities between strong and moderate binder for a given site, and there could be possibilities for suitably sized compounds to occupy multiple sites simultaneously. We further tried to probe these hypotheses by solving the appropriate drug bound crystal structures without any success. However we were able to crystallize rabbit skeletal calsequestrin (rCASQ1, PDB ID: 3V1W) in complex with a MPD. The amino acid sequences of CASQ1 and CASQ2 are highly conserved among human, canine and rabbit. Therefore we were able to qualitatively exploit the MPD binding profile of rCASQ1 crystal structure for evaluating the molecular docking result of canine crystal structure. In addition we were able to correlate these findings with the competitive inhibition of pharmaceutical drug binding by CAF and EGC, which was studied via molecular docking and isothermal calorimetry.

#### 2.2.1. Prediction of Binding Pockets for Different Classes of Drugs—Molecular Docking

**Figure 3:**
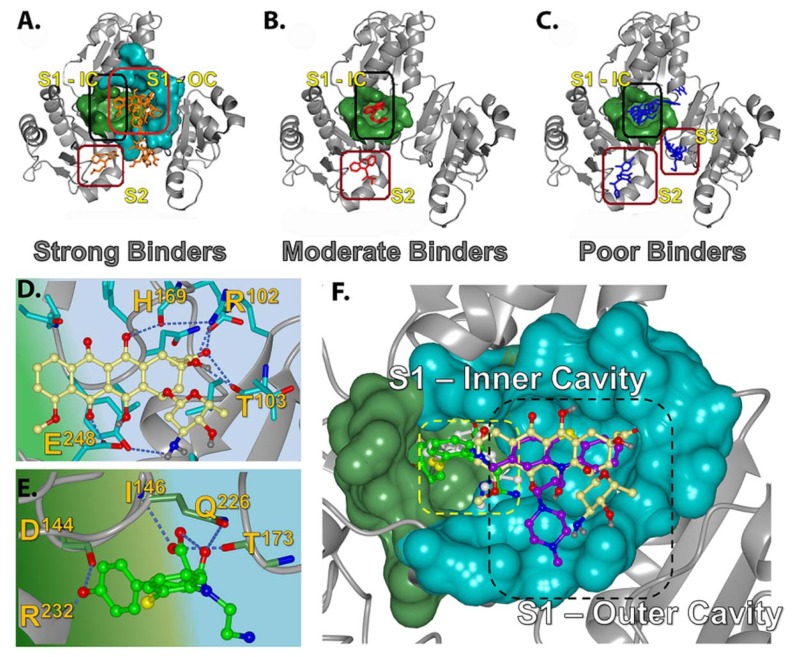
Binding-pockets predicted for the drug molecules. The different cavities and sub-cavities are highlighted by boxes with different colors and are individually labeled. (**A**) Strong binders; (**B**) Moderate binders; (**C**) Poor binders; (**D**) The predicted binding mode of doxorubicin (DOX) reveals its occupation at the larger S1-OC, being strengthened by several H-bonds with the neighboring amino acids; (**E**) The predicted binding mode of diltiazem (DTZ) reveals a unique orientation in which it occupies both the S1-IC (phenyl ring) and the S1-OC; (**F**) Two boxes with dashed lines indicate different regions within the S1 site. S1-IC is indicated by the yellow line and S1-OC is indicated the black line. These figures were generated using Open-Source PyMOL™ (v1.4).

The docking algorithm predicted that a large pocket located in the central junction, and a few other cavities located in the inter-domain junctions are the only sites accessible to drug molecules. We classified these sites as the S1, S2 and S3 binding pockets (Figure 3A–C). The S1 pocket encloses junctional cavity formed between the three domains of CASQ, and is comprised of an inner narrow sub-cavity (S1-IC; Figure 3F) and a larger outer cavity (S1-OC; Figure 3A–C,F). These cavities are formed by the amino acids Q^145^, I^146^, Y^173^, I^174^, Q^226^, R^227^, S^228^, R^231^, R^232^, L^233^, R^234^, D^237^, T^241^ (S1-IC); and I^104^, E^105^, D^107^, F^170^, P^172^, R^227^, R^231^, E^243^, D^244^ (S1-OC). The S2 pockets (Figure 3A–C) are cavities present in the thioredoxin-like folds of each domain, and are comparatively smaller than the S1 pocket. Although there are three S2 pockets, one located in the thioredoxin-like fold in each of the three domains, they displayed significant variations in size and surface charge due to variations in amino acid compositions within these pockets. The S2 site of domain III, located in the *C*-terminus, revealed the presence of a bigger hydrophobic pocket with long stretch of highly conserved amino acids, while that of domain I, located in the *N*-terminus, was short and made up of only 8 amino acids. Of these, only the S2 sites of domain I and domain III were found to be preferred by the studied drugs. The S3 sites are the interfacial crevices formed between domains I, II and III (Figure 3A–C), but only one of them were predicted to host a few poor binding molecules; none were preferred by any strong or moderate binders. The S2 sites in domains I & III are formed by the amino acids: K^54^, S^94^, E^105^, F^106^, D^107^, G^108^, F^118^, E^243^ on domain I (S2-D1); M^202^, L^233^, M^238^, F^239^, W^242^, H^250^, I^251^, V^252^, A^253^, F^254^, A^255^, E^256^, I^287^, D^289^, D^290^, F^292^, P^293^, L^294^, W^299^, E^300^, T^302^, F^303^, I^305^, D^306^, L^307^, P^310^, Q^311^, I^312^, G^313^, V^314^, V^315^, N^316^, V^317^, D^319^, A^320^, D^321^, S^322^, W^324^ ondomain III (S2-D3). The amino acid composition of the S3 site predicted to be accessed by poor binders is: D^125^, P^126^, E^168^, H^169^; S3-III/II: E^199^, H^225^, R^227^, T^277^, P^280^, L^282^; S3-III/I: E^91^, F^106^, D^107^, G^108^, E^109^, V^114^, H^225^, R^227^, D^234^, M^238^, E^243^ (S3-II/I).

#### 2.2.2. Strong Binders

Molecular docking predicted that the strong binders, such as DAN and DOX, preferred to bind into the S1-OC site in their top ranking poses, wherein their bulky aromatic rings were able to fit into the larger cavity and are stabilized by π-stacking. In addition, neighboring residues, H^169^, T^103^, and R^102^ established multiple hydrogen bonds, significantly strengthening their affinity for the S1 site relative to the moderate and poor binders (Figure 3D,E). On the other hand, the strong binders, such as AMD and VPM, preferred the S2 site (Figure 3A), while TDZ was predicted to bind into S1-IC in its top-ranking conformation. The docking scores for those strong binders correlated well with (*R*^2^ = 0.80) the corresponding experimental *K*_d_’s determined by ITC studies [9]. The common binding mode observed in the top ranking conformations of DAN, DOX, and TFP (Figure 3F) suggested that they might have similar electrostatic charge distributions and orientation of the steric bulk, which were in good agreement with the previously published quantum mechanics data calculated using minimal basis sets without any polarizable functions [8]. We also compared the charge distributions of the optimized structures of the predicted S1 pocket binding drugs DAN, TDZ and TFP (strong binders) with DTZ (moderate binder) calculated using polarized basis sets, which is discussed later. A good overlap of top ranking conformations and charge distributions of DAN, TDZ, and TFP adds strength to the prediction that the S1-OC is the most preferred pocket for known highly toxic pharmaceutical compounds.

#### 2.2.3. Moderate Binders

The moderate binders AMT, DTZ, NTP, and PMT were predicted to preferentially bind into S1-IC with a nice overlap of their aromatic rings in their top-ranking poses (Figure 3B). This is also supported by the fact that these molecules have very similar experimentally determined *K*_d_ values [9]. Visualization of docked results highlighted that the S1-IC pocket was more preferred by these compounds than the S1-OC due to their smaller aromatic ring sizes, compared to the bigger rings possessed by strong binders like DAN and DOX. Moreover, these molecules had fewer functional groups and were not suited to bind into polar S1-OC cavity. DTZ was unique among the moderate binders in that its benzyl ring occupied the S1-IC site and the thiazepine moiety occupied the S1-OC (Figure 3E), indicating that when two loosely connected aromatic rings are present, one of them goes into the S1-IC and the excess bulk is pushed into the bigger S1-OC cavity. Among such two ring systems, the one with polar functional groups tend to go into the polar S1-OC cavity, thus providing better opportunities for stabilization via H-bonded interactions. This hypothesis is supported by the fact that the thiazepine moiety of DTZ was predicted to form multiple hydrogen bonds with I146, Q226 and T173, while the other moderate binders like AMT, NTP, and PMT showed no hydrogen bonding. The placement of the thiazepine moiety in S1-OC resembled the common binding mode observed in strong binders, but, unlike DOX, DAN, and TFP, the DTZ it did not exclusively utilize the S1-OC, explaining its ability to achieve affinity in the range of strong binders like DAN and DOX. Other moderate binders, IMP and NTP, were also predicted to occupy the S1-IC, while AMT and PMT preferred either S2-DI or S2-DIII sites (Figure 3B). The reduced toxic side effects by these molecules suggest that stabilizing the S2 sites does not affect the biological function of CASQ as strongly as stabilizing the S1-OC by DAN and DOX.

#### 2.2.4. Poor Binders

Four of the five tested poor binders, EPH, IPT, MNZ, and MPL, preferred to bind either into one of the S3 pockets or S2-DII (Figure 3C), while HPD was predicted to bind to S1-IC (Figure 3C). They did not reveal any preference towards the polar S1-OC, which explains their poor binding nature due to lack of H-bonds. Nevertheless, the docking scores of those poor binders revealed higher scores in the range of moderate or strong binders and showed poor correlation with experimentally determined *K*_d_’s, which is possibly because of limitations in the docking method. It was previously shown at one instance that a basic amine group in some drug compounds did not formed any H-bonded interactions in the crystal structure, but molecular docking favorably top ranked the poses in which these groups formed H-bonded interactions with binding site residues [23]. Such circumstances lead to scoring bias and result in false positives with higher scores, as could have happened with our poor binding compound set.

### 2.3. Quantum Mechanics Studies of Ligand Charge Distributions: Comparison between Strong Binders Predicted to Bind into the S1-OC of hCASQ2

**Figure 4:**
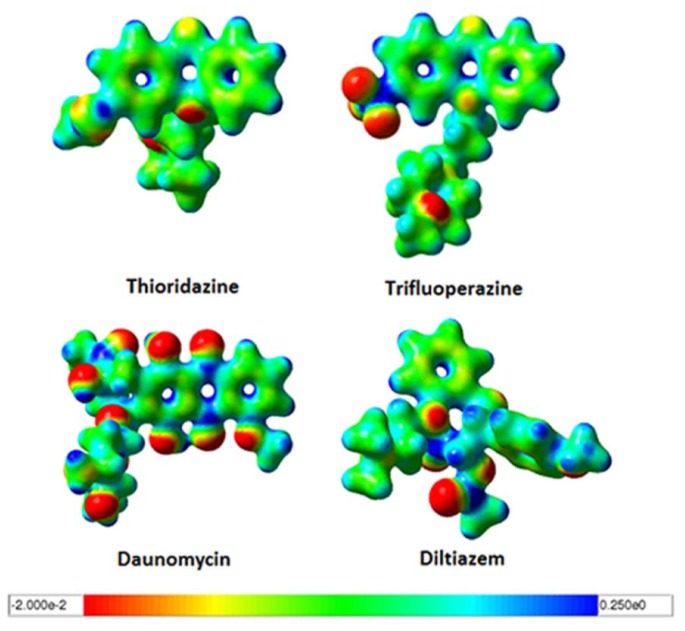
The iso-density surface maps for predicted hCASQ2S1-OC binders using the density functional theory method employed in Gaussian 03 [24]. The red and blue areas correspond to electron-rich (negative) and electron-poor (positive) regions, respectively. The color bar located at the bottom represents the charge scale.

The low micro molar range [9,10] binding affinities of strong binding pharmaceutical drugs: DAN, DOX and TFP in CASQ2 have been reported and are well known for their cardiotoxic side effects. We wanted to know if the charge distribution pattern between the predicted S1-OC binding drugs are similar and in accordance to the previously published data [8]. To accomplish this, we performed accurate quantum mechanical (QM) calculations for the strong binding drug molecules: DAN, TFP, TDZ; and a moderately binding molecule: DTZ in order to compare their optimized geometries and charge distribution properties (Figure 4). The QM calculations revealed that the strong binders TDZ and TFP had planar phenothiazine moieties that were highly neutral in the core. The side chains of TDZ and TFP showed variations with some polar atomic substitutions. But, the surface of DAN revealed that the core anthracycline ring was neutral in accordance with the observations in TDZ and TFP, but highlighted plenty of terminal negatively charged functional groups. This infers that all these drug molecules involve bulky hydrophobic interactions for a tight binding, but the DAN could produce both hydrophobic and polar interactions. The structure of DTZ revealed difference in the out-of-plane aromatic rings, where the methoxy phenyl ring can rotate flexibly, as it happened in the optimized structure (Figure 4 bottom right). These results displayed the similarities in electrostatic charge distributions between them, which in turn explained the docking predictions that they all bind into an identical pocket.

### 2.4. Amino Acid Conservation Analysis of Potential Small Molecule Binding Pockets in CASQ

**Figure 5:**
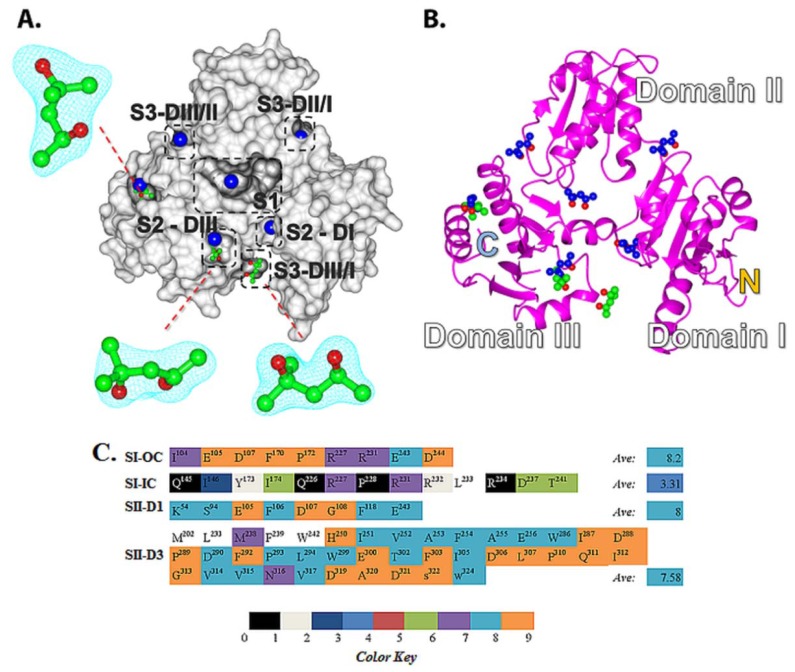
Binding sites for Drug Molecules: Evidences from crystallography and predictions from molecular docking. (**A**) Surface representation of the rCASQ1-MPD complex crystal structure. The three crystallographic 2-methyl-2,4-pentanediol (MPD) molecules are represented by ball and stick (green for carbon atoms and red for oxygen atoms). The corresponding electron density maps for MPD molecules were contoured at 1.5 σ level and are represented by sky blue mesh. The cavities accessible to MPDs as predicted by docking are represented by the blue balls; (**B**) Ribbon diagram representing the sites accessible for MPD molecules in the crystal structure of rCASQ1 (red and green) and for the predicted MDP molecules (blue and red). These figures were generated using Open-Source software PyMOL™ (v1.4); (**C**) Analysis of the sequence conservation of rCASQ1. The evolution of each amino acid forming the potential binding sites are individually scored by the server ConSurf. The average conservation scores are displayed at the far right hand side end, representing the average for each of the predicted binding site.

Amino acid sequence conservation profile of rCASQ1 was studied using the ConSurf [25–27] algorithm as employed in the web server. The target sequence (rCASQ1) was used for multiple alignments with other homologous CASQs that were retrieved separately using the BLAST [24] algorithm. The conservation profiles of amino acids at each position were later quantified in the score range of 1 (no conservation) to 9 (fully conserved) by ConSurf. This analysis revealed that at least 60%–70% of the whole CASQ sequence is conserved among a wide range of species, while >90% of the pockets that were predicted to host the known sets of strong, moderate and poor binding drugs (*i.e.*, S1 and S2) are highly conserved (Figure 5C). Since these two sites represent the junctions between the three domains of CASQ and can significantly affect the polymerization of CASQ. The overall conservation of amino acid sequences and both the SI-OC and SII pockets indicate that they are significant to the function of the protein and hence its normal function will be disturbed upon drug-association.

### 2.5. Structural Basis of 2-Methyl-2,4-pentanediol (MPD) Binding into rCASQ1: Crystal Structure Reveals Simultaneous Multiple Binding into S2-DIII Pocket Predicted by Molecular Docking

Several initial attempts to crystallize CASQ1 or CASQ2 in their drug-bound form did not succeed in the presence of drugs like DOX, DAN or TFP. However our attempt to co-crystallize rCASQ1 in complex with MPD, a molecule much smaller than the studied set of pharmaceutical drugs, generated a diffraction quality crystal (Figure 5A,B, PDB ID: 3V1W). The small and aliphatic nature of MPD presents it a high level of conformational flexibility and hence we expected it to simultaneously occupy a number of different surface pockets inrCASQ1. The crystal structure clearly revealed the positions for three MPD molecules in agreement with our hypothesis of simultaneous multiple drug binding (Figure 5A,B). All three MPD molecules were found localized within the S2-DIII domain at the *C*-terminus region of CASQ that is highly hydrophobic in nature. This crystal structure provided an initial idea about possible small molecule binding pockets.

### 2.6. Development of a Structure Model for Competitive Inhibition of Pharmaceutical Drugs by Caffeine (CAF) and Epigallocatechin (EGC)

#### 2.6.1. CAF/EGC Mediated Competitive Inhibition—Molecular Docking Studies

**Figure 6:**
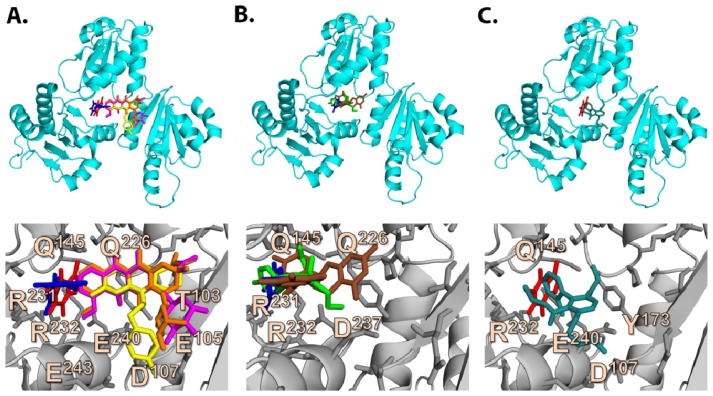
The structural insights for competitive inhibition. (**A**) Overlap of the binding modes for caffeine (red)/gallocatechin (blue) and strong binders; daunorubicin (magenta), doxorubicin (brown), and trifluoperazine (yellow); (**B**) Overlap of the binding modes for diltiazem (green), epigallocatechin (blue), and epigallocatechin gallate; (**C**) Overlap of the binding modes for amlodipine (teal) and caffeine (red). These figures were generated using Open-Source PyMOL (v1.4).

**Figure 7:**
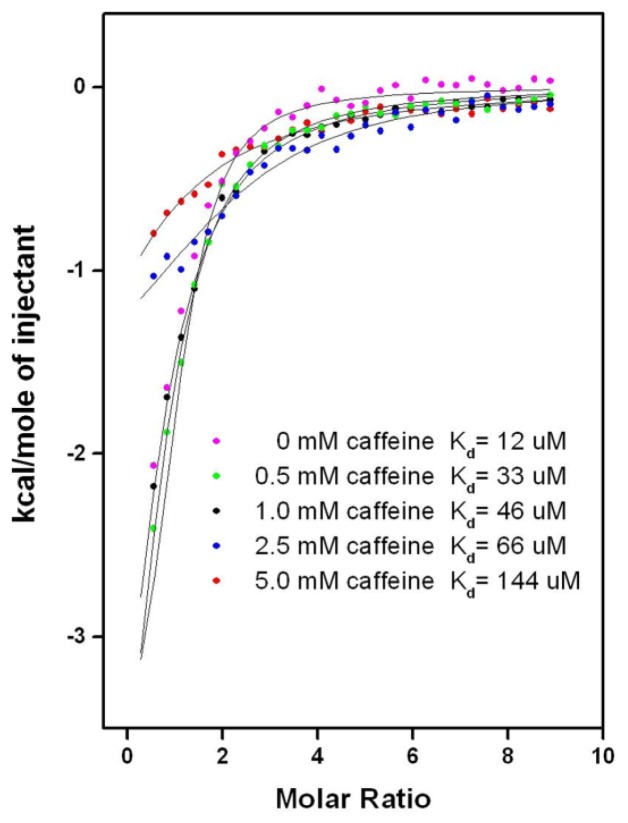
Comparison of the heat released upon amlodipine binding to hCASQ2 in the presence of 0 (pink), 0.5 (green), 1.0 (black), 2.5 (blue) and 5 mM (red) caffeine concentrations. The corresponding *K*_d_ values for amlodipine are indicated.

We further developed a structure model to understand the CAF and/or EGC mediated antagonistic activities [8,22] against drug-induced muscular toxicities at the molecular level. To accomplish this, we docked the CAF, EGC and EGCG into CASQ by applying the same docking approach as the pharmaceutical drugs. The predicted binding sites for CAF and EGC from the top ranking binding poses were then compared with those of strong and moderate affinity drugs to check for any overlap. As expected, there were direct structural overlaps between CAF, EGC, and most of hCASQ2-affinity drugs in their binding sites (Figure 6A,C), suggesting a plausible mechanism for CAF/EGC-mediated competitive inhibition. The docking scores for CAF and EGC were ~−3 and ~−6 kcal·mol^−1^, indicating a loose binding that correlated to poor and moderate binding range at S1 and S2 cavities, respectively. The top scoring pose of CAF revealed binding into the S1 pocket, while energy difference for it to bind into a S2 pocket was ~1 kcal·mol^−1^. However, EGC was predicted to bind into S2 cavity in its highest-ranking conformations and the energy difference was ~0.1 kcal·mol^−1^ for it to bind into the S1 pocket. Such low penalties associated with occupying one or other pockets suggested possibilities for the simultaneous binding in more than one cavity for these natural compounds, in which case, all drug molecules that bind either to S1 and S2 sites would be antagonized by them. The smaller number of aromatic rings and the hydrophobic nature seemed to allow CAF to fit snugly into the S1-IC, but the size of its aromatic core extends out of the S1-IC pocket. Thus, the docking results of CAF suggest a steric hindrance for strong binders like: AMD, DAN/DOX, and TFP to be accommodated in S1-OC (red molecule in Figure 6A,C, Figure 7, and Supplemental file 2). The predicted CAF binding mode leads to: (i) the extension of bulky aromatic core into the S1-OC cavity, and (ii) a high possibility of binding pocket rearrangement because of the observed tight fit of CAF core into the static crystal structure. Similarly, when the EGC bound into S1, it oriented in such a way that the trihydroxyphenyl ring occupied the S1-IC and extended its fused ring into the S1-OC in a manner highly similar to that of DTZ (Figure 6B), thus directly overlapping with the strong binding drug molecules. We further addressed the possibilities for simultaneous multiple binding of CAF and EGC by checking their inhibitory activities that prevent the association of drugs that were predicted to bind inS1 and S2 cavities via ITC assays.

#### 2.6.2. CAF/EGC Mediated Competitive Inhibition—Isothermal Calorimetry Assays

We performed ITC assays to check the predicted competition between the natural compounds (CAF, EGC, and EGCG) and tested synthetic drugs. However, addition of EGC or EGCG to the buffer instantly precipitated the other components, limiting the inhibition studies to be carried for CAF only (Figure 7 and Supplemental files 2, 3). Among all of the studied drugs, the solubility of amlodipine was least affected by CAF, making it the most amenable drug for use in ITC competitive-inhibition assays. As shown in Figure 7, a series of titrations containing 50 μM hCASQ2 solutions pre-incubated with 0, 0.5, 1.0, 2.5, and 5 mM of CAF showed a gradual reduction in the *K*_d_ of amlodipine from 12 to 33, 46, 66, and 144 μM, supporting the computational prediction that CAF inhibits drug-binding to CASQ2. Although inhibitory effect of CAF to the strong and moderately binding drugs were established at a 5 mM CAF concentration, the concentration dependency could not be established for many other drug molecules due to unfavorable interactions with the solution that led to their precipitation. Data showing the inhibition by CAF for other CASQ2 affinity drugs are given in Supplemental file 2. Thus the ITC assays confirmed the docking prediction of competitive inhibition by showing significantly reduced affinity of all tested drug molecules to hCASQ2 in the presence of CAFITC is also suggestive of CAF binding into both S1 and S2 cavities simultaneously, as a result of which, the drugs predicted to bind into S1 and S2 cavities were successfully inhibited.

### 2.7. Implications of Drug Binding on the CASQ Function

**Figure 8:**
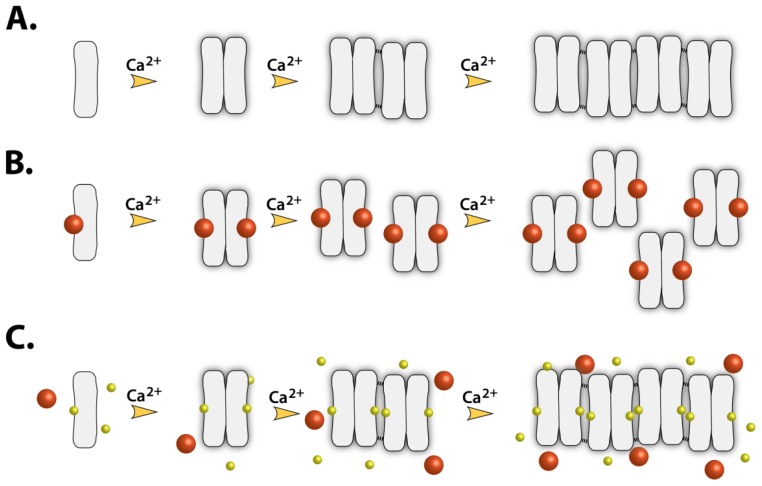
Schematic diagram of the polymerization of CASQ2. (**A**) The mechanism of Ca^2+^-sequestration via polymerization of the CASQ monomers; (**B**) The hypothetical inhibition mechanism of drug preventing polymerization of CASQ. The drugs are represented as big orange spheres; (**C**) In the presence of high concentrations of weak binders such as CAF and EGC (small yellow spheres), occupation by strong binders is being inhibited, while the weakly binding small molecules do not significantly affect CASQ2 polymerization.

We have previously shown that strong- and moderate-affinity CASQ-binding drugs significantly disrupt polymerization of CASQ (Figure 8) and consequently, its high capacity Ca^2+^-binding [9,18]. Our previous data with TFP indicate that Ca^2+^ is being released from SR microsomes upon its diffusion into SR [10,11] probably due to its association with CASQ and increased free Ca^2+^ concentration inside the SR above a threshold value of RyR channel opening, where similar sized hydrophobic control drugs showed no effect on Ca^2+^ release [10,11]. Moreover, TFP was shown to significantly reduce the total SR microsomal Ca^2+^ content in a concentration- and time-dependent manner [10,11]. As the predicted drug binding pockets, S1 and S2, are located at the dimer interface of CASQ, we hypothesize that the association of drugs into either of these sites would significantly affect the interfacial geometry and consequently disrupt the polymerization of CASQ (Figure 8-B), which is a very crucial mechanism in the SR Ca^2+^ regulation. In addition, altered polymerization caused by drug binding could change CASQ’s affinity to the Ca^2+^-release channel complex proteins such as RyR, triadin, and junctin, and, consequently, affect the opening probability of RyR2, although it is still highly controversial which oligomeric form of CASQ2 interacts with RyR2 [6,28,29]. Also substantial amount of polymorphisms have been known in CASQ2 sequence and its post-translational modifications [14,30]. This led to a general notion that certain individuals might be more sensitive to certain drugs than others in terms of muscle toxicity. It is tempting to speculate that some of those polymorphisms of CASQ could further increase the affinity of CASQ to certain drugs or their metabolites due to the altered characteristics of their binding pockets.

## 3. Experimental Section

### 3.1. Preparation of Native Rabbit Calsequestrin (rCASQ1)

Extraction of rCASQ1 from isolated SR microsomal was performed as previously described [10], but with an increase from 5 mM to 20 mM imidazole in buffer A and addition of 0.05% (*w*/*v*) NaN_3_. The SR microsomal extract was dialyzed into buffer B (20 mM MOPS, 0.5 M NaCl, 1 mM EGTA, 0.05% NaN_3_, pH 7.2) and applied to phenyl-sepharose resin (GE Healthcare). After washing the resin extensively with buffer B, rCASQ1 was eluted using buffer C (20 mM MOPS, 0.5 M NaCl, 15 mM CaCl_2_, pH 7.2, 0.05% (*w*/*v*) NaN_3_), then buffer exchanged into buffer D (5 mM sodium phosphate, 0.05% NaN_3_, pH 6.8) and applied to a CHT-10-I hydroxyapatite column (BioRad, CA) equilibrated with the same buffer. rCASQ1 was then eluted from the column with a linear gradient from 60%–100% buffer E (500 mM sodium phosphate, pH 6.8). Hydroxyapatite elution fractions containing rCASQ1 were pooled and exchanged into crystallization buffer (20 mM HEPES, 0.5 M NaCl, pH 7.0) and concentrated for crystallization.

### 3.2. Crystallization of rCASQ1 and Structure Refinement

Crystals of native rCASQ1 were grown at 4 °C using the vapor diffusion method by mixing protein at a concentration of 10 mg·mL^−1^ in crystallization buffer with an equal volume of reservoir buffer (0.1 M HEPES, 0.2 M NaCl, pH 7.0) containing 27.5% (*v*/*v*) 2-methyl-2,4-pentanediol (MPD) as the precipitating agent. Small crystals developed overnight and fully developed in one to two weeks. All crystals were of the orthorhombic space group C222_1_ with one molecule in the asymmetric unit. Diffraction data were collected at the Advanced Light Source (Beamline 8.2.1) and reduced and scaled using HKL2000. Iterative adjustments and refinements were performed using COOT and PHENIX [31], respectively, using the deposited coordinates of native rCASQ1 (PDB ID: 3TRQ). The final coordinates have been deposited to the RCSB (PDB ID: 3V1W), and were used for docking studies of MPD-binding sites. Complex crystallizations of CASQ from various species with several compounds such as daunomycin, doxorubicin, verapamil, diltiazem and trifluoperazine were attempted, but none diffracted well enough to be pursued.

### 3.3. Iso-Density Surface Calculation

Gas phase geometry optimizations using the B3LYP hybrid functional [32–34] and 6-31 + g(d) basis sets were performed in Gaussian 03 [35] in order to calculate the surface charge distribution for daunomycin (DAN), desipramine (DSP), diltiazem (DTZ), propafenone (PFN), thioridazine (TDZ), and trifluoperazine (TFP). The resultant electrostatic potentials were mapped on their van der Waals surfaces (Figure 1) using GaussView 3 [36]. The surface charge distribution of drugs with similarly-charged surfaces were cross-compared to determine if they bound in a likewise manner in docking experiments.

### 3.4. Isothermal Titration Calorimetry (ITC)

ITC experiments were conducted using a previous protocol [9], with slight modifications. The protein and drug concentrations were kept at 50 μM and 2 mM, respectively, while the CAF concentration was allowed to vary between 0 and 5 mM for different drugs. Compounds with poor aqueous solubility were dissolved first in DMSO and then diluted into ITC buffer with final concentrations of DMSO not exceeding 2%. Origin software (Origin Lab Corp, Northampton, MA, USA) was used to fit the data to a one-site binding model via non-linear least squares regression.

### 3.5. Computational Molecular Docking

All molecular docking simulations were performed using AutoDock 4.0 [37]. Ligand molecules were either retrieved from the NCBI PubChem web server [38] or generated with the Accelrys Discovery Studio Visualizer3.0 [39]. Any van der Waals clashes were cleared before final use. The ligand bond angles, bond distances, and dihedrals were treated as flexible in the docking process, while the protein was treated as a rigid receptor. Finally, these structures were converted into PDBQT format by AutoDock Tools [40], allowing only the polar hydrogen atoms to be present in docking. As the drug-binding site(s) in CASQ2 had not yet been established, blind docking for all drug molecules was performed by enclosing the whole protein inside a large grid box, to include all possible sites in the search. The atomic affinity grids were calculated using the AutoGrid module of AutoDock4.0. Default parameters were used, except those for torsional rotations, which were reduced from 50° to 25° in order to increase the exhaustiveness of the search. A total of 100 docking runs were performed for each drug molecule using the Lamarckian Genetic Algorithm, resulting in 100 best conformations. Finally, the pool of predicted binding orientations were subjected to cluster analysis with a 2 Å root-mean-square deviation cut-off, whereby the conformations within each cluster were ranked in order of increasing docking scores, which is a measure of the free energy of binding.

## 4. Conclusions

We report on the structural basis for drug-affinity of hCASQ2 by comparing molecular docking predictions with experimentally identified binding sites of multiple MPD ligands in the crystal structure of rCASQ1. Our approach revealed possible binding pockets in hCASQ2 for known toxic pharmaceutical drugs. In addition, our molecular docking results indicated the possibility for simultaneous binding of multiple EGC and CAF molecules in more than one pocket, which was supported by the observation of multiple MPD-binding in the rCASQ1 crystal structure. The known drug molecules were sorted into three different groups based on their docking affinities to hCASQ2 as strong, moderate, and poor binders. Three different binding cavities: S1, S2, and S3 emerged as the binding sites for those compounds. Of these three sites, the polar outer cavity of S1 (S1-OC) was predicted to be the most preferred target for highly toxic drugs like DAN, DOX and TFP due to their bulky aromatic rings and opportunities for multiple hydrogen bond interactions with their polar terminal groups. However, these physico-chemical characteristics were relatively reduced in the moderately binding drugs. The known poor binding drugs had highly flexible side chains with several charged groups but were predicted to occupy the hydrophobic S2 or S3 sites due to smaller sizes of aromatic cores. Especially the most crucial difference seen in the poor binders were significant reduction in the size of their bulky aromatic rings. The CAF and EGC were predicted to bind loosely due to their smaller aromatic cores into the same binding pockets where the known highly toxic drugs were predicted to bind. Thus these natural compounds could compete with the drug molecules for the same binding pocket. These were consistent with our ITC data showing CAF mediated competitive inhibition of drugs binding to CASQ emphasizing the accuracy of our structure model.

## Supplementary Materials



## Supplementary Materials
