# Supplementary material for: Molecular Mechanisms of Pharmaceutical Drug Binding into Calsequestrin
Source: Int J Mol Sci. 2012 Nov 6;13(11):14326–43. doi: 10.3390/ijms131114326 (PMC3509583; doi:10.3390/ijms131114326)
Supplement: Supplementary file 1 [file ijms-13-14326-s001.pdf]

Supplement Data 1

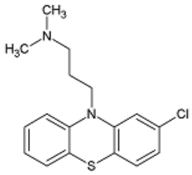

Chlorpromazine

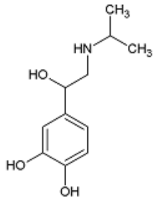

Isoproterenol

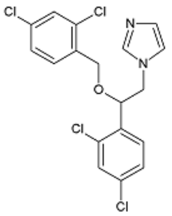

Miconazole

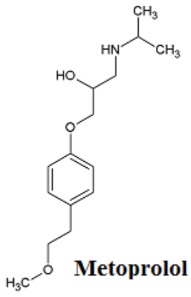

Metoprolol

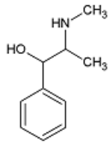

Ephedrine

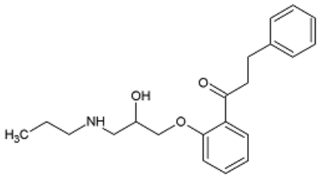

Propafenone

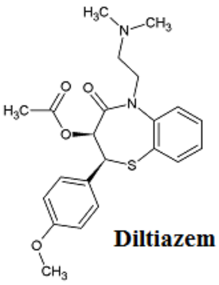

Diltiazem

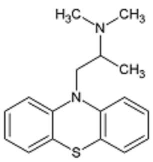

Promethazine

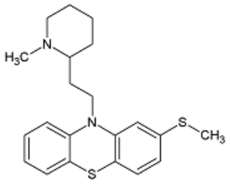

Thioridazine

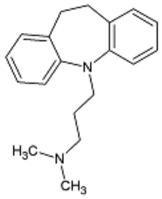

Imipramine

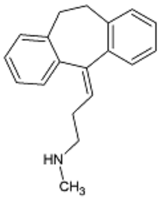

Nortriptyline

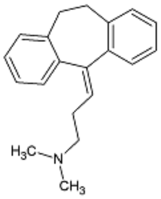

Amitriptyline
