# Supplementary material for: Molecular Mechanisms of Pharmaceutical Drug Binding into Calsequestrin
Source: Int J Mol Sci. 2012 Nov 6;13(11):14326–43. doi: 10.3390/ijms131114326 (PMC3509583; doi:10.3390/ijms131114326)
Supplement: Supplementary file 2 [file ijms-13-14326-s002.pdf]

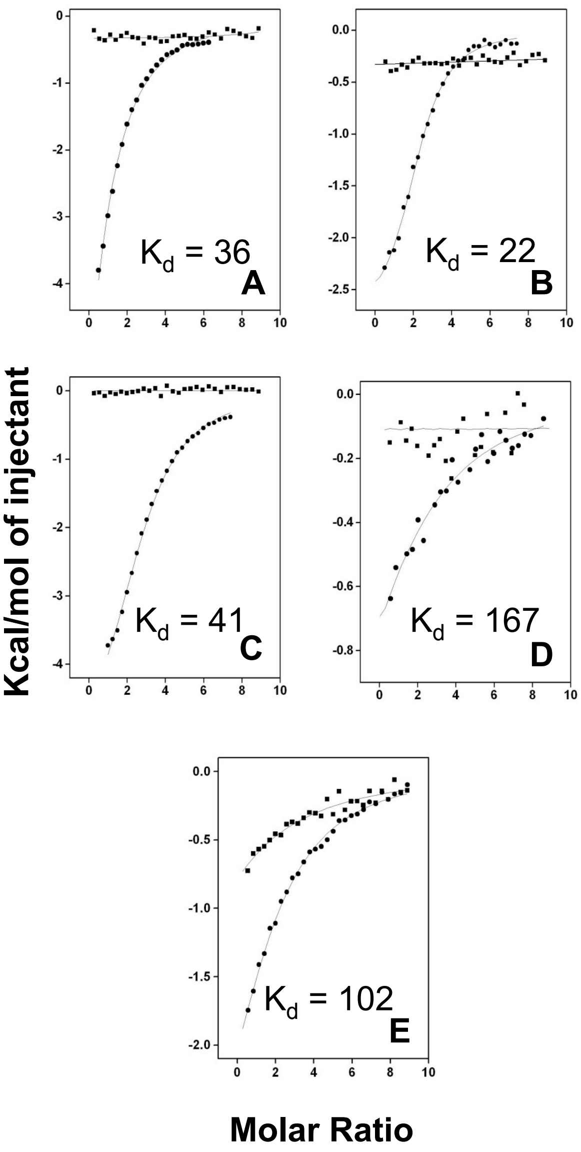

Supplemental Fig. 2 Effect of caffeine on binding of various drugs by hCASQ2. Protein was titrated in the presence of a constant 5 mM concentration of caffeine. A) trifluoperazine, B) thioridazine, C) diltiazem, D) imipramine and E) chlorpromazine. Dissociation constants are for the titration in the absence of caffeine and is expressed as μM.
